# Nano-Magnesium Silicate Hydroxide/Crumpled Graphene Balls Composites, a Novel Kind of Lubricating Additive with High Performance for Friction and Wear Reduction

**DOI:** 10.3390/ma13173669

**Published:** 2020-08-19

**Authors:** Tong Zhang, Jianguo Zhao, Jin Zhang, Shanshan Zhang, Jingwei Li, Shijie Li, Xinyu Li, Jie Zhang

**Affiliations:** 1 Institute of Carbon Materials Science, Shanxi Datong University, Datong 037009, China; 15735298432@163.com (T.Z.); shanzhangss@163.com (S.Z.); bjyddx1988@163.com (J.L.); li841974@sina.com (S.L.); chunyemen509@163.com (X.L.); zhangjie798554896@126.com (J.Z.); 2 Institute of Chemistry and Materials Science, Shanxi Normal University, Linfen 041000, China; 3 Key Laboratory of Coal Science and Technology of Ministry of Education and Shanxi Province, Taiyuan University of Technology, Taiyuan 030024, China

**Keywords:** crumpled graphene balls, magnesium silicate hydroxide, lubricating oil, tribological properties

## Abstract

In this study, crumpled graphene balls (CGB), a kind of nano-material, was used as an additive to improve the tribological properties of base oil. Nano-magnesium silicate hydroxide (MSH)/CGB composites were prepared by ultrasound-assisted liquid-phase exfoliation. The loading of MSH significantly increased the number of pleats and reduced the lamellar thickness of CGB. Then, in order to improve the compatibility with the base oil, the MSH/CGB composites were decorated with oleic acid and stearic acid to get modified lipophilic composites (ML-MSH/CGB). The ML-MSH/CGB were characterized by Fourier transform infrared spectroscopy (FTIR), X-ray diffraction (XRD) and scanning electron microscopy (SEM). In addition, the tribological properties of the ML-MSH/CGB in base oils were investigated using a ball-on-disc setup tribometer. It indicated that the fantastic tribological behavior of the ML-MSH/CGB in base oil may contribute to a smaller and extremely wrinkled laminated structure. Furthermore, the base oil with 0.005 wt% ML-MSH/CGB composites exhibited the best anti-friction effect, and its average friction coefficient, wearing capacity and wear scar diameter were reduced by 25.4%, 22.1% and 16.7%, respectively. The introduction of ML-MSH/CGB composed materials is an excellent strategy to optimize the friction performance of lubricating oil.

## 1. Introduction

Friction and wear have a huge impact on aspects of energy, the environment, technology and the economy, all over the world. More importantly, energy consumption is usually accompanied by the release of carbon dioxide. Therefore, the utilization of lubricating oil is particularly indispensable [1]. Lubricating oil reduces friction between contacting surfaces and thus increases the energy efficiency of engines and other machines. They can also reduce wear, thereby extending the life of tribological components. The most effective way to improve the friction performance of lubricating oil is by adding excellent additives to the base oil. However, traditional lubricant additives such as MoS_2_, polytetrafluoroethylene (PTFE) and zinc dialkyldithiophosphate (ZDDP) can lose their tribological properties at elevated temperatures [2,3,4]. Moreover, lubricating oil that includes S and P elements can cause severe environmental pollution [5]. Compared with traditional lubricant additives, ultrafine particles are appealing by virtue of their size and their chemical and thermal stability under tribological conditions. Ultrafine particles not only maintain their excellent tribological properties in high temperatures, but also can better repair the worn parts of machinery. Nano-additives include graphene [6], calcium borate [7], nano-soft metals such as copper [8], organic borides [9,10] and so on. However, it is challenging to disperse ultrafine particles in lubricating oils.

Graphene, a kind of nano-material, has attracted tremendous attention because of its excellent tribological properties, such as thermal stability, high strength, smooth surface as well as low shear strength [11,12,13]. Recent research has shown that graphene has improved the friction performance of worn surfaces tremendously [14,15]. However, aggregation is a major problem for graphene additives in lubricant oil because it reduces the effective particle concentrations, prevents graphene from entering the contact area of working surfaces, and leads to unstable tribological performance, which greatly limits its further utilization [16,17]. In order to obtain homogeneously dispersed graphene in base oil, special treatment for graphene is necessary. Crumpled graphene ball (CGB), a special kind of graphene with a rough surface structure, was first reported to be used as a lubricating oil additive in 2016 and can significantly improve the lubrication properties of lubricant oil. [18,19,20,21,22]. In detail, CGB changes the surface contact of graphene to point contact, thereby reducing the coagulation of graphene [21]. Xuan Dou et al. found that the dispersibility of the CGB in base oil (PAO_4_) is much more stable than other carbon-based materials, e.g., graphite, r-GO and carbon black [22]. In addition, CGB is better than commercial lubricants in reducing friction and wear, but there are some disadvantages of CGB; for example, the structure of CGB is easily destroyed under friction conditions and gradually fails.

In order to further improve the friction properties of CGB in lubricating oil, an excellent modification strategy is to introduce nanoparticles into the lamellar structure of CGB. In the process of synthesizing magnesium silicate hydroxide (MSH)/CGB with the liquid-phase ultrasonic stripping method, MSH peels the CGB into smaller fragments. In this process, MSH may act as nano-ball bearings, so that the graphene fragments form a more wrinkled and curled structure. Therefore, in the process of friction, modified lipophilic composites (ML)-MSH/CGB, which are smaller and more wrinkled, easily fill the potholes of the friction surface and repair the friction surface, at the same time as reducing friction and wear [23]. Magnesium silicate hydroxide (MSH) is the main component of serpentine minerals, which display excellent friction reduction, compression resistance and self-repair. It is easy to form an oxidation protective layer with good wear resistance and high hardness on the surface of friction couple, so it can be used as an oil additive [24,25]. In this work, MSH nanoparticles were synthesized by a one-step hydrothermal method, using nano-SiO_2_ and MgO as raw materials. Then, a new kind of CGB (MSH/CGB) was prepared by loading nano-MSH onto the surface of CGB. Furthermore, in order to improve the compatibility with the base oil, the MSH/CGB were decorated with oleic acid and stearic acid to get modified lipophilic composites (ML-MSH/CGB). The material properties of ML-MSH/CGB were characterized. In addition, the tribological properties of ML-MSH/CGB in base oils were investigated using a ball-on-disc setup tribometer. Remarkably, the base oil with just 0.005 wt% of the ML-MSH/CGB has shown a significant anti-friction effect.

## 2. Materials and Methods

### 2.1. Materials

Base oil SN500 was purchased from Hebei Yuansite Petrochemical Co., Ltd. (Xingtai, China). The chemicals including stearic acid and n-butyl alcohol were provided by Tianjin Hedong District Hongyan Reagent Factory (Tianjin, China). NaOH was obtained from Tianjin Sailboat Chemical Reagent Technology Co., Ltd. (Tianjin, China). Oleic acid was supplied by Tianjin Yatai United Chemical Co., Ltd. (Tianjin, China). Sulfuric acid (H_2_SO_4_, 98%) was purchased from Hebei Guan Yongfei Chemical Factory (Langfang, China). Nano-MgO was obtained from Shanghai Keyan Industrial Co., Ltd. (Shanghai, China). Nano-SiO_2_ was provided by Chengdu Huaxia Chemical Reagent Co., Ltd. (Chengdu, China). Hydrazine hydrate was obtained from Aladdin Chemical Co., Ltd. (Shanghai, China). All chemicals used were of analytical reagent grade.

### 2.2. Preparation of CGB and Nano-MSH

Firstly, graphene oxide (GO) was prepared through the modified Hummers method [26,27]. GO suspension with 8 g·L^−1^ was ultrasonicated for 1 h, and then subjected to sufficient spray drying to obtain brown-yellow GO powders [28]. The test conditions for spray drying are heating temperature 180 °C, peristaltic pump power 5% and fan power 55%. After that, part of the brown-yellow GO powders was calcined in a muffle furnace preheated to 300 °C to obtain black crumpled graphene balls. As can be seen from the color change, GO was reduced partially [28,29].

Nano-MSH was synthesized by a one-step hydrothermal method. The reaction was carried out by adding appropriate quantities of nano-MgO and nano-SiO_2_ into deionized water (mass ratio 1:1), and then the pH was adjusted to 13 with 6 mol·L^−1^ NaOH aqueous solution. After mixing and stirring for 1 h, the solution was transferred to the autoclave. The reaction was carried out at 200 °C for 12 h (rotate speed 450 r·min^−1^). Finally, the nano-MSH powders were obtained after suction filtration, washing and vacuum drying.

### 2.3. Preparation and Modification of Nano-MSH/CGB Composites

MSH/CGB composites were prepared via the liquid-phase ultrasonic stripping technique. MSH and CGB were totally dissolved in deionized water with mass ratios of 1:1, 5:1, 10:1. The reaction temperature was kept at 60 °C under vigorous stirring. After 1 h, it was conducted to the ultrasonic stripping for 5 h. The product was washed repeatedly with deionized water and freeze-dried to obtain nano-MSH/CGB composites.

A certain amount of oleic acid and stearic acid was ultrasonically dispersed in 60 mL n-butanol. A given mass of MSH/CGB complex was added to the above solution with the aid of ultrasonic for 30 min, and a few drops of concentrated sulfuric acid were added dropwise. After reacting at 100 °C for 8 h under magnetic stirring, hydrazine hydrate was added and the temperature was reduced to 80 °C. After reacting for 10 h, the reaction mixture was washed with anhydrous ethanol and suction filtration was performed. Finally, the obtained residue was named the modified lipophilic MSH/CGB composites (ML-MSH/CGB). Meanwhile, the CGB was subjected to lipophilic modification according to the above modification method to obtain modified crumpled graphene ball (MCGB).

### 2.4. Preparation of ML-MSH/CGB Oil

Five parts of SN500 base oil (kinematic viscosity 108 mm^2^·s^−1^ at 40 °C) with 50 g were weighed equally at first, and then 1:1 ML-MSH/CGB, 5:1 ML-MSH/CGB, 10:1 ML-MSH/CGB, MSH and MCGB powders with 0.02 wt% of base oil were accurately introduced into the base oil. Next, 0.02 wt% oleic acid surfactant was added to promote the dispersion of the powders in the lubricating oil. After that, the oil samples were placed in the ultrasonic cell grinder (Yuming Instrument Co., Ltd., Shanghai, China), and the ultrasonic power was 300 W for 3 min. Finally, the five kinds of ML-MSH/CGB base oil were transferred to the ball-on-disc setup tribometer (Hengxu Testing Machine Technology Co., Ltd., Jinan, China) to test the friction performance.

Through the above friction test, the ratio of MSH to CGB with the best anti-friction performance can be obtained. The optimum addition amount was explored, the ML-MSH/CGB powders with 0.005 wt%, 0.01 wt%, 0.015 wt%, 0.02 wt% and 0.025 wt% of base oil were added to the base oil, respectively. The other processes were the same as above-mentioned. A few samples were transferred to ampere flasks, labeled and placed at room temperature. The dispersion of ML-MSH/CGB in oil samples was observed and recorded regularly. In the end, the remaining samples were transferred to the ball-on-disc setup tribometer for the friction and wear test.

### 2.5. Tribological Properties and Structural Characterization of ML-MSH/CGB Composites as Oil Additive

The microstructure of the samples was characterized by a field-emission scanning electron microscope (SU8020, Hitachi High-tech Co., Ltd., Tokyo, Japan). Chemical compositions and crystallite structures of the as-obtained composites were examined by XRD (Rigaku D/max-2550, Rigaku Corporation, Tokyo, Japan) using Cu Kα radiation from 10 to 70 angles. X-ray photoelectron spectra (XPS) were obtained by a VG scientific ESCALAB 250 spectrometer (Thermo Fisher Technology Co., Ltd., Waltham, MA, USA). The infrared (IR) spectra were performed on a Bruker IFS 66 V/S Fourier transform infrared spectroscopy (FTIR) spectrometer using KBr pellets (Bruker Technology Co., Ltd., Beijing, China). Raman spectra were recorded on an inVia Qontor laser confocal Roman spectrometer with 532 nm laser excitation (Siruiwei Technology Co., Ltd., Beijing, China).

A ball-on-disc tribometer was used to study the tribological properties. A friction performance test was based on the industry standard SHT-0762-2005 (lubricant friction coefficient measurement method) and carried out at a sliding velocity of 0.4 m/s and under a fixed load of 50 N for 60 min; a room temperature of 22 °C was maintained throughout the whole test process and the number of cycles was 22920. The 5 mm diameter steel balls were made of GCr15A bearing steel and the lower plates were NM400 wear-resistant steel plates with a diameter of 20 mm, a thickness of 3 mm and a hardness of 400 HB. Before the test, the steel balls, steel plates and fixtures were cleaned ultrasonically by petroleum ether. Next, 10 mL oil samples were added to the steel plates, and then dripped according to the immersion degree of the steel balls (to ensure that the liquid level of the oil samples exceeds at least 3 mm on the top of the steel balls) in line with the tribometer.

### 2.6. The Measurement of Wear Scar Diameter (WSD) and Wearing Capacity (WC)

The diameter of the wear scar was measured under scanning electron microscopy (SEM), and each sample was measured three times. Before the friction experiment, the mass of the small steel ball is called m_1_. After the friction experiment, the mass of the small steel ball is m_2_, and the wear capacity can be obtained according to the following formula:

Wearing capacity = m_1_ − m_2_

## 3. Results and Discussion

### 3.1. Structure and Morphology

**Figure 1:**
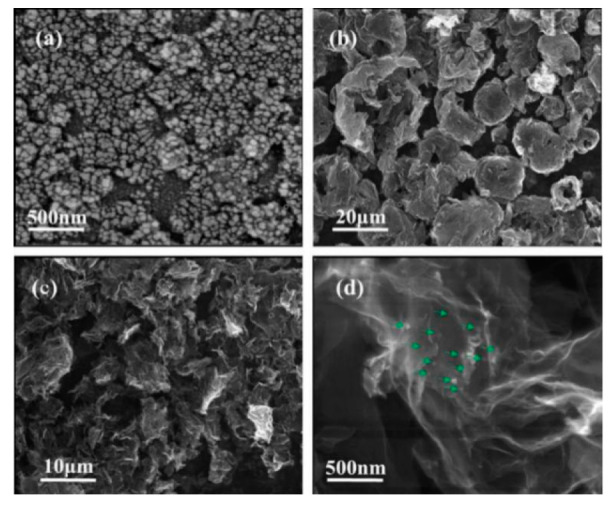
Typical low and high-magnification scanning electron microscopy (SEM) images of (**a**) nano-magnesium silicate hydroxide (MSH) particles, (**b**) crumpled graphene balls (CGB), (**c**,**d**) MSH/CGB composites.

The micro-topography of as-obtained MSH, CGB and MSH/CGB composites are investigated by means of field-emission scanning electron microscopy (SEM). As shown in Figure 1a, the average diameter of MSH spherical particles is about 50 nm. Figure 1b displays the micromorphology of CGB, which is an irregular pleated globular structure with a particle size of approximately 10–20 μm. When CGB and MSH are combined with the aid of ultrasound, it is found that the irregular pleated globular structure is opened to form smaller, thinner and more wrinkled graphene structure (Figure 1c), wherein the MSH nanoparticles are uniformly attached to the surface of the crumpled graphene (Figure 1d). It is suggested that MSH/CGB composites are successfully synthesized by the liquid-phase ultrasonic stripping method, which prevents the agglomeration of graphene effectively and is more prone to enhance the tribological properties in worn surfaces.

**Figure 2:**
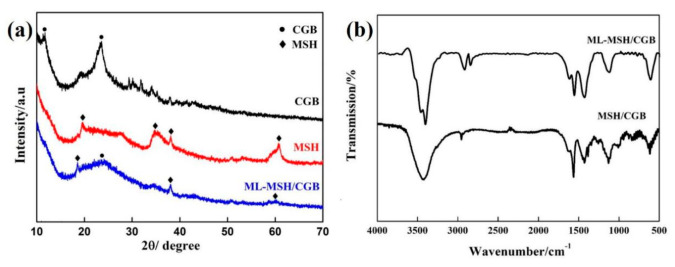
(**a**) X-ray diffraction (XRD) spectrum of CGB, MSH and modified lipophilic composites (ML)-MSH/CGB, (**b**) Fourier transform infrared spectroscopy (FTIR) patterns of MSH/CGB and ML-MSH/CGB.

Figure 2a is the XRD spectrum of CGB, MSH and ML-MSH/CGB. The characteristic diffraction peak of the graphene oxide in CGB locates at 2θ = 11.74°, indicating that CGB is not fully reduced via thermal reaction [30]. For MSH nanoparticles, the corresponding diffraction peaks are located at 2θ = 19.57°, 35.14°, 37.48°, and 60.62°, which can be assigned to (110), (132), (202), and (029) crystal faces, respectively. It belongs to a kind of fiber serpentine (in agreement with Powder Diffraction File (PDF) card 25-0645) [31]. As for the ML-MSH/CGB, the diffraction peak located at 24.15° belongs to CGB and the diffraction peaks at 18.76°, 37.91°, and 59.99° derive from MSH. Apparently, MSH particles with a diameter of 50 nm are distributed triumphantly on the surface of CGB. Furthermore, since the -COOH contained in oleic acid and stearic acid reacts with the unreduced oxygen-containing functional groups on the CGB, the disappearance of the characteristic diffraction peak of CGB further indicates that MSH/CGB was successfully subjected to lipophilic modification. In order to further explore the structural changes of graphene oxide during the reaction, the Raman test was performed on the sample and the result is shown in Figure S1. The Raman spectrum of GO, ML-MSH/CGB and ML-MSH/CGB after friction reveals a G band at ~1580 cm^−1^ deriving from the characteristic peak of the in-plane vibration of a layer of sp2 carbon atoms. The peak at around ~1353 cm^−1^ can be assigned to the D band. Therefore, judging from the peak position in the figure, the structure of graphene oxide does not change after the MSH particles are loaded on the surface of the graphene oxide, the lipophilic modification is performed, and then the friction test is carried out.

Figure 2b displays the infrared spectra of MSH/CGB and ML-MSH/CGB. The peak at 1630 cm^−1^ refers to C=C bonds. The absorption peak at 3426 cm^−1^ in MSH/CGB spectra corresponds to O-H stretching vibration, which subsequently supposes a possibility of lipophilic modification [32]. There appear some fresh peaks in the spectrum of the ML-MSH/CGB in comparison to that of MSH/CGB. The bands at 2926 cm^−1^ and 2842 cm^−1^ are assigned to the stretching vibration of -CH2 and -CH3, respectively [33]. This indicates that long-chain alkanes were successfully grafted to the surface of MSH/CGB.

### 3.2. Tribological Properties of ML-MSH/CGB in Base Oil

**Figure 3:**
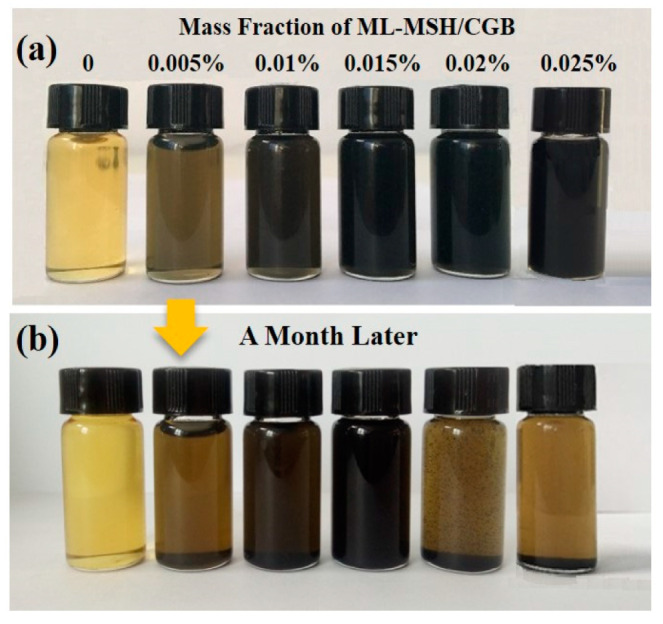
Dispersion photographs of ML-MSH/CGB oil with different contents (**a**) immediately after sonication and (**b**) a month after sonication.

The dispersibility of as-prepared ML-MSH/CGB oil with different contents (0.005, 0.01, 0.015, 0.02 and 0.025 wt%) is measured right after sonication (Figure 3). The lubricating oil of 0.015 wt% additive experiences no aggregation after a month of standing at room temperature, confirming that the as-obtained ML-MSH/CGB additive is of good oil solubility in lubrication.

**Figure 4:**
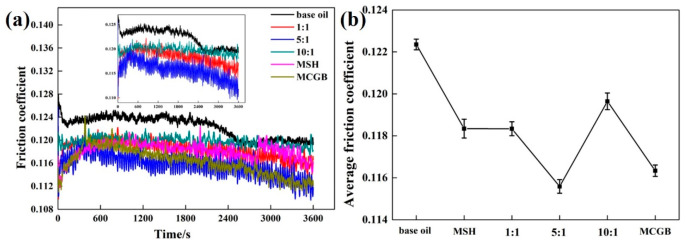
(**a**) The friction coefficient curves of ML-MSH/CGB oil with different proportions depending on friction time; and (**b**) average friction coefficient of ML-MSH/CGB oil as a function of proportion.

Figure 4a displays the friction coefficient of ML-MSH/CGB base oil with different ratios of MSH to CGB over time. The mass ratio of MSH to CGB is 1:1, 5:1 and 10:1 respectively, and the addition of ML-MSH/CGB in base oil is 0.02 wt%. From the figure, it can be seen that the friction coefficient of ML-MSH/CGB base oil shows a tendency to increase short-term and then gently decrease with the increase in test time, but they are all smaller than pure base oil. Among ML-MSH/CGB oil with different proportions, the friction coefficient of 5:1 base oil is the lowest, showing the best anti-friction effect. Too little MSH cannot break down the pleated graphene ball sufficiently, while too much MSH may increase the roughness of the surface and increase the friction coefficient. Therefore, the appropriate ratio of MSH to CGB will maximize the friction performance. In order to clearly see the change of friction coefficient with proportion, the average value of the friction coefficient of each proportion is calculated as shown in Figure 4b. It can be seen that the average friction coefficient (AFC) of oil with different proportions is 10:1 > 1:1 > pure MSH > pure MCGB > 5:1 from high to low. The average friction coefficient of pure MSH and pure MCGB oil is decreased by 3.4% and 4.9% compared with pure base oil, respectively. The average friction coefficient of 1:1 and 10:1 ML-MSH/CGB oil is higher than that of pure MSH and pure MCGB base oil, while the average friction coefficient of 5:1 ML-MSH/MCGB oil is obviously lower than that of pure MSH and pure MCGB oil. It is 5.4% lower than pure base oil, which shows a better synergistic anti-friction effect.

**Figure 5:**
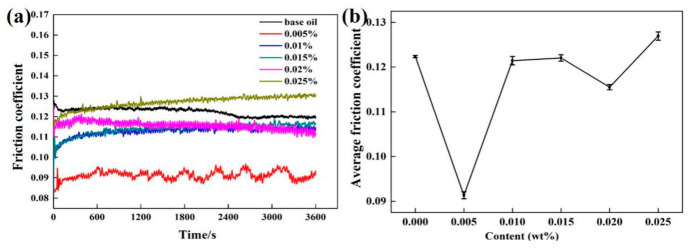
(**a**) The friction coefficient curves of ML-MSH/CGB oil with different addition amounts depending on friction time; and (**b**) average friction coefficient of ML-MSH/CGB oil as a function of added content.

It can be seen from the above results that the ML-MSH/CGB oil with a mass ratio of 5:1 has a good anti-friction effect, so the friction coefficient of different addition amounts of ML-MSH/CGB in base oil is investigated, and the result is shown in Figure 5. The friction coefficient of ML-MSH/CGB oil with different additions shows a relatively stable trend with the increase in test time (inset of Figure 5a). Among the five different mass fractions of ML-MSH/CGB oil, the friction coefficient of 0.025 wt% addition is higher than that of pure base oil, and the friction coefficient of 0.005 wt% addition is much lower than that of pure base oil. The possible reason for this is that it is difficult to form a strong friction film with insufficient composites, while excessive composites will lead to accumulation and increase the roughness of the friction surface. Therefore, adding the appropriate amount of ML-MSH/CGB composites into base oil can improve its friction performance, of which 0.005 wt% is the most significant. The corresponding curve of average friction coefficient is shown in Figure 5b. The average friction coefficient (AFC) displays the rule of decreasing first and then increasing. The friction performance of SN500 becomes worse when too little or too much ML-MSH/CGB is added, hence an optimum addition amount can improve the friction performance of the SN500 base oil. Among them, the AFC of 0.005 wt% ML-MSH/CGB oil is 0.0912 and decreases by 25.4% when compared with pure base oil.

**Figure 6:**
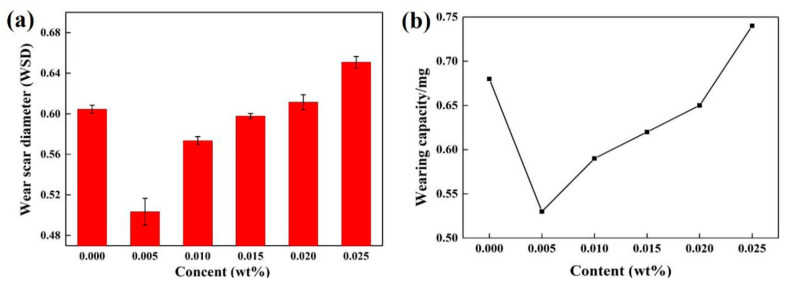
Wear scar diameter (**a**) and wearing capacity (**b**) of ML-MSH/CGB oil as a function of added content.

The wear scar diameter (WSD) and wearing capacity (WC) after the steel ball friction test are also another parameter to measure the friction and wear situation (Figure 6). Obviously, there is a remarkable decrease in the WSD and WC value upon increasing content in oil. Both the WSD and WC value of the ML-MSH/CGB lubricant reach its minimum value at 0.005 wt%, which decline by about 16.7% and 22.1% compared to those of the base oil. Meanwhile, it can be seen that the ML-MSH/CGB composites, used as an additive, play a positive role in reducing the friction and wear of oil.

**Figure 7:**
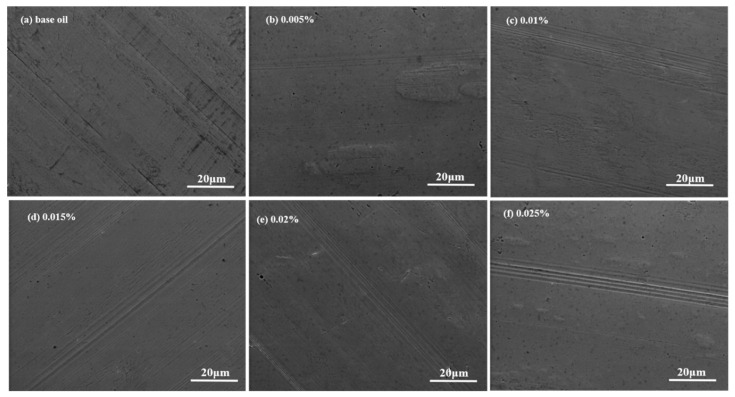
SEM images of the wear scar on the surface of the steel ball after friction in the oil samples with different additions of ML-MSH/CGB: (**a**) base oil (**b**) 0.005 wt% (**c**) 0.01 wt% (**d**) 0.015 wt% (**e**) 0.02 wt% (**f**) 0.025 wt%.

After the friction tests, the surface morphology of each ball wear spot is observed, and the SEM images are shown in Figure 7. The wear condition is measured mainly from the number of ploughs, the depth and width of wear marks on the surface. Firstly, it can be clearly seen that the surface wear scar of SN500 base oil is wider and more obvious. After adding ML-MSH/CGB, the wear scar is obviously narrowed, but the number of furrows is obviously increased, of which the furrows of 0.015 wt% and 0.02 wt%, 0.025 wt% ML-MSH/CGB oil is more obvious. It is clear that the wear degree of ML-MSH/CGB oil with 0.005 wt% and 0.01 wt% is relatively shallow. The surface wear of 0.005 wt% ML-MSH/CGB oil is the smallest, and the anti-friction and wear performance is the strongest. This demonstrates that ML-MSH/CGB with smaller size and larger fold degree is more likely to enter into the surface of the friction pair to form a lubrication film to enhance the anti-friction performance of lubricating oil.

**Figure 8:**
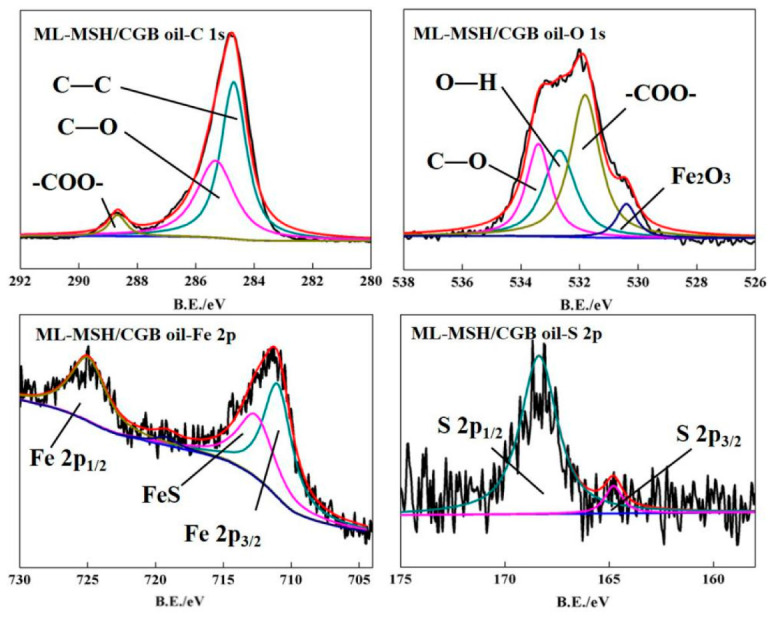
XPS spectrum of steel ball surface after friction test of ML-MSH/CGB oil.

**Figure 9:**
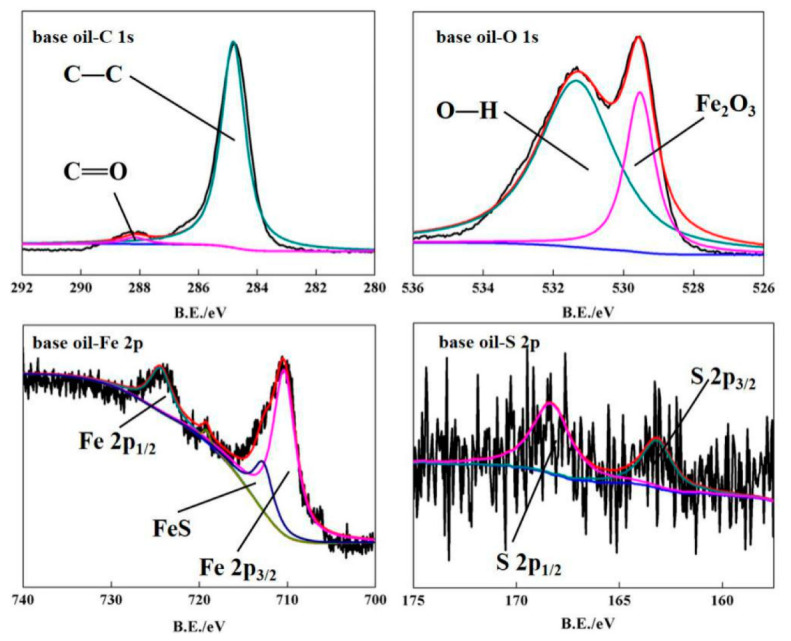
XPS spectrum of steel ball surface after friction test of SN500 base oil.

In order to explore the possible reasons for the excellent friction reduction and compression resistance of oil with ML-MSH/CGB powers, the element composition and valence bonds of steel ball surface after friction test of ML-MSH/CGB oil are analyzed and tested, and the experimental results are shown in Figure 8. From the XPS spectra of O 1s and Fe 2p elements, it can be seen that the worn surface contains compounds such as Fe_2_O_3_ and FeS, indicating that reaction films are formed on the surface of steel ball in the friction process to protect the friction surface. Compared with the XPS spectra of base oil (as shown in Figure 9), the new functional groups such as C-O and -COO- appear in the XPS spectrum of C 1s and O 1s elements, indicating that ML-MSH/CGB composites attach to the wear surface in the friction process.

## 4. Conclusions

A novel kind of MSH/CGB composites have been successfully prepared by the liquid-phase ultrasonic stripping method and modified with oleic acid and stearic acid. Adding modified MSH/CGB composites to base oil can obtain excellent friction and anti-wear properties. Compared with base oil, the average friction coefficient, wearing capacity and wear scar diameter of the as-prepared MSH/CGB oil are reduced by 25.4%, 22.1% and 16.7%, respectively. The possible reason for this is that MSH peels the CGB into smaller fragments under the liquid-phase ultrasonic stripping technology, and uses itself as the nano-spherical bearings, which makes the graphene fragments more wrinkled, thereby achieving the effect of reducing friction and wear. It is easier to enter the friction surface to form a lubrication film to protect the friction pair surface. Therefore, this new type of CGB with self-dispersion and mechanical properties is an attractive material for tribological applications.

## Supplementary Materials

The following are available online at https://www.mdpi.com/1996-1944/13/17/3669/s1. Figure S1. The Raman spectrum of GO, ML-MSH/CGB and ML-MSH/CGB after friction.
