# Supplementary material for: Nano-Magnesium Silicate Hydroxide/Crumpled Graphene Balls Composites, a Novel Kind of Lubricating Additive with High Performance for Friction and Wear Reduction
Source: Materials (Basel). 2020 Aug 19;13(17):3669. doi: 10.3390/ma13173669 (PMC7503855; doi:10.3390/ma13173669)
Supplement: Supplementary file 1 [file materials-13-03669-s001.pdf]

materials

Supplementary Information

Nano-Magnesium Silicate Hydroxide/Crumpled Graphene Balls Composites, a Novel Kind of Lubricating Additive with High Performance for Friction and Wear Reduction

Tong Zhang [1,2], Jianguo Zhao [1,2,*], Jin Zhang [1,*], Shanshan Zhang [1,3], Jingwei Li [1], Shijie Li [1], Xinyu Li [1] and Jie Zhang [1]

[1] Institute of Carbon Materials Science, Shanxi Datong University, Datong 037009, Shanxi, China; 15735298432@163.com (T.Z.); shanzhangss@163.com (S.Z.); bjyddx1988@163.com (J.L.); li841974@sina.com (S.L.); chunyemen509@163.com (X.L.); zhangjie798554896@126.com (J.Z.)

[2] Institute of Chemistry and Materials Science, Shanxi Normal University, Linfen 041000, Shanxi, China

[3] Key Laboratory of Coal Science and Technology of Ministry of Education and Shanxi Province, Taiyuan University of Technology, Taiyuan 030024, Shanxi, China

* Correspondence: jgzhaoshi@163.com (J.Z.); zhangjin50@hrbeu.edu.cn (J.Z.); Tel.: +86-13133329150 (J.Z.); +86-13734209435 (J.Z.)

Received: 19 June 2020; Accepted: 10 August 2020; Published: date

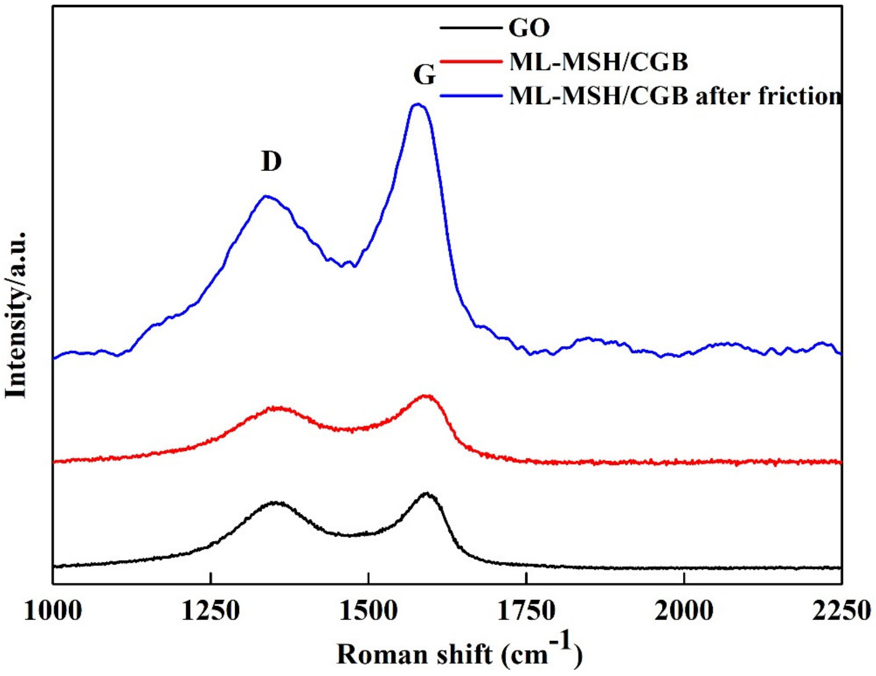

Figure S1. The Raman spectrum of GO, ML-MSH/CGB and ML-MSH/CGB after friction.
